# Sound level intensity severely disrupts sleep in ventilated ICU patients throughout a 24-h period: a preliminary 24-h study of sleep stages and associated sound levels

**DOI:** 10.1186/s13613-017-0248-7

**Published:** 2017-03-03

**Authors:** Maxime Elbaz, Damien Léger, Fabien Sauvet, Benoit Champigneulle, Stéphane Rio, Mélanie Strauss, Mounir Chennaoui, Christian Guilleminault, Jean Paul Mira

**Affiliations:** 1 Centre du Sommeil et de la Vigilance, Hôtel-Dieu de Paris, APHP, Université Paris Descartes, Paris, France; 2 EA 7330 VIFASOM Sommeil-Vigilance-Fatigue et Santé Publique, Hôtel Dieu de Paris, Université Paris Descartes, 1 place du Parvis Notre Dame, 75004 Paris, France; 3 Unité Fatigue et Vigilance, Institut de Recherche Biomédicale des Armées (IRBA), Brétigny-sur-Orge, France; 4 Cognitive Neuroimaging Unit, U992, INSERM, Gif/Yvette, France; 5 Service de Réanimation médicale, Hôpital Cochin, APHP, Université Paris Descartes, Paris, France; 6 NeuroSpin Center Institute of Bioimaging, Commissariat à l’Energie Atomique (CEA), Gif/Yvette, France; 7 Stanford University Sleep Disorders Clinic, Palo Alto, CA USA

**Keywords:** Intensive care unit, Weaning, Sleep, Sound intensity, Monitoring

## Abstract

**Background:**

It is well recognized that sleep is severely disturbed in patients in intensive care units (ICU) and that this can compromise their rehabilitation potential. However, it is still difficult to objectively assess sleep quantity and quality and the determinants of sleep disturbance remain unclear. The aim of this study was therefore to evaluate carefully the impact of ICU sound intensity levels and their sources on ICU patients’ sleep over a 24-h period.

**Methods:**

Sleep and sound levels were recorded in 11 ICU intubated patients who met the criteria. Sleep was recorded using a miniaturized multi-channel ambulatory recording device. Sound intensity levels and their sources were recorded with the Nox-T3 monitor. A 30-s epoch-by-epoch analysis of sleep stages and sound data was carried out. Multinomial and binomial logistic regressions were used to associate sleep stages, wakefulness and sleep–wake transitions with sound levels and their sources.

**Results:**

The subjects slept a median of 502.2 [283.2–718.9] min per 24 h; 356.9 [188.6–590.9] min at night (22.00–08.00) and 168.5 [142.5–243.3] during daytime (8 am–10 pm). Median sound intensity level reached 70.2 [65.1–80.3] dBC at night. Sound thresholds leading to disturbed sleep were 63 dBC during the day and 59 dBC during the night. With levels above 77 dBC, the incidence of arousals (OR 3.9, 95% CI 3.0–5.0) and sleep-to-wake transitions (OR 7.6, 95% CI 4.1–14) increased. The most disturbing noises sources were monitor alarms (OR 4.5, 95% CI 3.5–5.6) and ventilator alarms (OR 4.2, 95% CI 2.9–6.1).

**Conclusions:**

We have shown, in a small group of 11 non-severe ICU patients, that sound level intensity, a major disturbance factor of sleep continuity, should be strictly controlled on a 24-h profile.

## Background

Having a sufficient amount of sleep during a 24-h period is generally recommended as a major means of promoting health in adults [1, 2]. Sleep has a crucial role in many somatic, cognitive and psychological processes, and “sleeping well” appears to be a health imperative [3, 4]. Increasing evidence shows that sleeping too little impacts severely on health with an increasing risk of morbidity [5] and even mortality [6] across many groups. It may also affect memory and immunity, and can jeopardize safety [7, 8]. Chronic short sleep duration (<6 h) has been associated with an increased risk of obesity, diabetes, hypertension and other cardiovascular diseases [1, 9, 10]. Acute sleep deprivation (defined as sleeping 25–50% of a normal 8 h night’s sleep) contributes to increased inflammation and disturbs the immunological response [11].

Patients hospitalized in intensive care units (ICU) are likely to require support of two or more organ systems, including respiratory support. Such patients would clearly benefit from an optimal environment. However, patients often complain that their ICU stay was severely disturbed by noise, light and an inadequate environment to rest and sleep. Poor sleep is considered a major concern in ICU because of its potential interaction with other psychological and somatic diseases and its impact on rehabilitation [12–14]. However, previous studies have been limited at least by three major concerns:

- The quality of polysomnographic sleep recording (PSG) was traditionally considered poor due to interference from mechanical ventilators and alarms [12, 15–17] prior to the recent introduction of new technologies such as the ActiWave.
- There was an absence of precise analyses of how sound peaks may be linked to sleep arousals. Sound levels have been measured very accurately in ICU. However, few have made definitive assessments of potential associations between the overall level of sound (sound intensity) or sound peaks, and sleep arousals. This is principally because there are a large number of sound peaks in the environment and ICU patients experience many sleep arousals.

The goal of this study was therefore:

- To specifically characterize and classify the different sources of sounds peaks and significant sound intensity levels disturbing sleep.
- To analyze sleep quality on a 24-h basis.

## Methods

### Subjects

The eleven patients enrolled in this observational study were under mechanical ventilation and responded to the following inclusion criteria: resolution of disease for which the patient was intubated, cardiovascular stability with no need of vasopressors, no sedation for at least three days and adequate oxygenation defined as *P*aO_2_/FiO_2_ of at least 150 mmHg with positive end-expiratory pressure (PEEP) up to 8 cmH_2_O. Exclusion criteria included a reported history of sleep disorders, including obstructive sleep apnea syndrome (OSAS) or insomnia, a diagnosed neurological disorder, renal insufficiency and any treatment with psychotropic drugs. The recruitment period was 16 months, and the data were collected over 7 months.

Patients were informed of the sleep protocol, received a written protocol and gave their informed consent to the study. Recording anonym data from the Cochin ICU patients has been approved by the National Ethical Data Protection Committee (CNIL) according to ethical committee procedures.

### ICU

This study identifies that these particular ICU sound levels are above the limit recommended by the WHO and result in a higher incidence of disturbance of sleep patterns. Apart from the sound from alarms, other sounds coming from people around are implicated in the discussion of sleep continuity and quality.

The ICU department studied comprised 4 units of 6 beds with two nurses and one nursing auxiliary allocated to each. The nursing station was about 5 meters from the rooms. Patient were lying in bed the all day, but were mobilized by one medical intern and two physiotherapists (15 min each three times per day). Moreover, the ICU was open to visits regardless of time during the day and night with the median number of conversation noises per hour statistically higher during the day compared to the night. In emergencies, doctors and staff were called via a microphone system which may also disturb patients’ sleep. This study did not identify a significant impact of conversation noise on sleep disturbances, but limiting voice loudness level and the frequency of conversations held by the bed side could certainly be recommended.

No specific instructions for patient sleep support (e.g., ear plugs, eye masks) were given to staff at the time of the study.

The rooms were lit by natural light from one window and artificial light at ceiling level. Lights were switched off manually by staff at night, with no specific schedule but depending on care intensity. The doors of the rooms were continually open day and night. The ICU unit had been refurnished recently to attenuate sound levels in ceilings and floors, with large specialist glass panels in order to improve supervision of patients.

### Sleep recording and analysis

Sleep was recorded for 24 h, from 18.00 to 18.00 the following day, using a miniaturized multi-channel ambulatory recording device (ActiWave^®^, CamNtech Ltd England). This device collected three electroencephalogram (EEG) derivations (F1–M2, C3–M2 and O1–M2), two transversal electrooculogram (EOG) channels (E1–M2 and E2–M1) and one chin EMG channel. ActiWave is a miniaturized polysomnography device which is positioned directly onto the scalp using very short electrodes which eliminates interference in the signals. Three ActiWave devices were used, synchronized to within 1 ms and with the same time resolution criteria in order to get the final Polysomnographic file. This was validated against standard polysomnography of patients with a sleep disorder [18]. Bio-electrical signals were digitized at a sampling frequency of 200 Hz with a 16-bit quantization between −500 and 500 μV, within a bandwidth of 0–48 Hz. All the data were stored in computer files using the standard EDF data format. EEG and EOG cup-electrodes (Ag–AgCl) were attached to the scalp and to the right and left cantus of the subjects using EC2 electrode cream (Grass Technologies, An Astro-Med, West Warwick, USA) according to the international 10–20 system for electrode placement. Sleep technicians tested each electrode at the start for signal quality in order to ensure clarity and easy interpretation over the 24-h period. Sleep analysis was performed in 30-s epochs according to the American Academy of Sleep Medicine’s (AASM) [19] standardized rules for scoring sleep stages and classification of waking and sleep into five levels (awake, non-REM stage [N1, N2, N3] and REM sleep). Arousals were defined as “abrupt shifts in EEG frequency including alpha, theta and/or frequencies greater than 16 Hz (but not spindles) that lasted at least 3–15 s with at least 10 s of stable sleep preceding the change” [20]. Natural waking was also scored and included in the WASO (Wake After Sleep Onset) values.

### Sound levels and assessment of their sources

Sound levels and their sources were positioned 40 cm from the subject’s head and recorded simultaneously with PSG, using a microphone T3 device (NoxMedical^®^, Reykjavik, Iceland) with C-weighting (dBC) decibel calibrated sound. The Nox T3 signals were synchronized to the ActiWave montage signals by manual adjustment of the major sound pressure level events recorded by the Nox T3. The synchronized ActiWave signals were truncated to match Nox T3 recording times and fed through the Nox T3 software (Noxturnal 4.3) to output at 30-s intervals with sleep/wake and sound pressure levels.

Qualitative scoring of all sources of sound was done by two researchers listening continuously to each of the 24-h recordings obtained with the Noxturnal 3.2 software (NoxMedical^®^, Reykjavik, Iceland), and scoring was performed every 30 s. The most clearly identifiable sounds were classified into three main categories: monitor alarms, mechanical ventilator alarms and conversations. If none of these three categories was recognized, the sound was classified as “other.” If many different types of sound were monitored during a given period, the most prevalent source was the one selected by the researchers.

One major aim of our study was to understand which sources of sound intensity, and at which decibel levels, were associated with arousals and/or change in sleep stages in order to calculate “cutoff values” above which sound level intensity disturbed sleep with a 95% confidence interval. Sleep, sound intensity levels and sound qualitative scoring were millisecond-synchronized and scored using the Noxturnal 3.2 software (NoxMedical^®^; Reykjavik, Iceland).

### Statistics

Sleep and sound intensity sources were time-synchronized on the same software and analyzed epoch-by-epoch for each subject. For each 30-s period of sleep or waking, an average level of sound was calculated and the main source determined.

Statistical tests were performed using R studio (Version 0.99.175—© 2009–2014 RStudio, Inc.), and significance (α risk) was fixed at *p* < 0.05. Continuous variables were presented as mean ± standard deviation (m ± SD), and means were compared (day vs. night) using a paired 2-tailed *t* test. Dichotomous variables were presented as occurrence and percentage [*n* (%)]. A Chi-square was used to test the relationship between dichotomous variables. When significant, the odds ratio and its 95% confidence interval [OR (95% CI)] were calculated. Adjusted ORs (ORa) and 95% CI for subject, hour and age were also calculated. Dependence between quantitative variables was checked using a Pearson correlation test (*r* ≥ 0.6, *p* < 0.05). Dependence between quantitative and qualitative variables was tested using an analysis of variance (ANOVA). A logistic regression was performed to estimate the probability for binary outcome (arousal, waking and sleep stage transitions) with noise level. A value of *p* < 0.05 for the Wald criterion was considered to denote regression coefficients significantly different from zero. The results are shown as ORs with 95% CIs. The fit of the models was judged using the Likelihood Ratio Test Statistic. Non-binary (more than 2 categories) dichotomous variables were analyzed using multinomial logistic regression models whereby noise level and hour were entered to predict awake and sleep stage.

## Results

Eleven consecutive subjects were included in the study (patient’s characteristics are shown in Table 1). Five patients had been hospitalized for septic shock, 4 for acute respiratory failure, 1 for hemorrhagic shock and 1 after major surgical procedure.

**Table 1:** Patient characteristics

Characteristics	Value
Age, yrs	64.2 ± 13.6
BMI, kg m^−2^	29.2 ± 5.4
SAPSII score	74.3 ± 27.8
APACHE II score	30.1 ± 11.4
ICU LOS (days)	22.1 ± 18.5
MV duration (days)	15.2 ± 13.3

*Key*: yrs, years; BMI, body mass index; SAPSII, new simplified acute physiology score; APACHE II score, acute physiological score chronic health evaluation; ICU LOS, ICU length of stay; MV duration, mechanical ventilator duration

### Sleep recordings (Table 2)

The quality of sleep recording was excellent for all 11 subjects, and no data were excluded based on reciprocal analysis of sleep signal quality with a final analysis of 95 040 PSG epochs of 30 s. Undetermined epochs due to artifacts or loss of signal represented <0.2% of the total scoring.

**Table 2:** Sleep characteristics (11 subjects)

Sleep values	24-h night and day sleep	Day (08h00–22h00)	Night (22h00–08h00)	Day vs. night *p*
TST, min	502.2 [283.2–718.9]	168.5 [142.5–243.3]	356.9 [188.6–590.9]	0.001
N1, min	112.1 [41.2–155.1]	32.1 [6.9–38.8]	98.2 [29.3–123.1]	0.002
N1, %TST	22.3 [14.6–30.7]	17.3 [4.6–21.7]	21.5 [7.5–28.5]	NS
N2, min	315.2 [242.1–480.3]	125.5 [52.3–144.7]	233.0 [143.3–364.0]	0.002
N2, %TST	62.7 [54.2–77.3]	68.7 [64.2–78.3]	62.1 [55.4–69.6]	NS
N3, min	55.2 [0–81.5]	23.5 [0–81.5]	39.7 [0–98.8]	NS
N3, %TST	9.0 [0–19.7]	3.5 [0–22.6]	6.5 [0–23.6]	NS
REM, min	20.1 [0–41.1]	5.3 [0–15.1]	19.8 [0–0.38]	0.04
REM, %TST	4.0 [0–9.7]	1.5 [0–3.9]	3.9 [0–10.1]	NS
Arousals, *n*/h	20 [6–32.6]	2 [0–16.6]	20 [8–22.7]	0.03

Data are median [interquartile range], TST = total sleep time, REM = rapid eye movement sleep, N1, N2, N3 = non-REM slow wave sleep stage N1, N2 and N3

The median total sleep time (TST) over 24 h was 8.3 h (502 [283.2–718.9] min) with one-third of sleep (168.5 min [33%]) occurring during the daytime (i.e., 8 am–10 pm). REM sleep represented only 4% of TST during the 24 h and N3 sleep represented 9% of TST.

Percentages of N1, N2, N3 and REM sleep were not significantly different between night and day periods. But the median number of arousals per hour during the night was about tenfold those during the day (20 [6–32.6] vs. 2 [8–22.7]; *p* = 0.03).

### Sound intensity level assessments (Table 3)

**Fig. 1:**
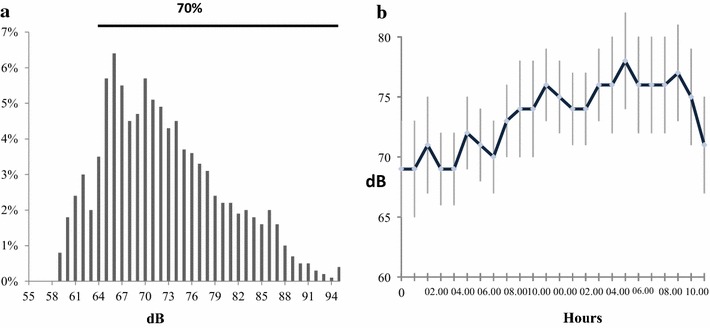
Sound during the 24-h period in ICU. *Key On the left*, the respective percentages of total sound occurrences per decibel unit between 59 and 95 dBC, are shown. 70% of noises occurrences are between 65 and 95 dBC. On the right, the average dBC level every 10 s during the 24-h period is shown

Sound characteristics, based on the analysis of 95 040 30-s epochs of recording per subject, are reported in Table 3 and Fig. 1.

**Table 3:** Sound characteristics (11 subjects)

Sound values	24-h night and day sleep	Day (08h00–22h00)	Night (22h00–08h00)	Day vs. night *p*
Sound level, dBC	72.2 [65.1–80.2]	74.2 [68.1–80.2]	70.2 [65.1–80.3]	0.01
Sound typology				
Alarm, *n*/h	38 [25–51]	22 [14–24]	18 [15–23]	NS
Mechanical respirators, *n*/h	101 [50–120]	55 [38–57]	49 [41–62]	NS
Talking; staff conversations, *n*/h	28 [10–53]	26 [9–43]	5 [4–15]	0.02
Other, *n*/h	15 [7–25]	10 [3–17]	7 [4–10]	NS

The median level and interquartile range of noise in the rooms was 72.2 dBC [65.1–80.2] and was higher during the day than at night.

Qualitatively, the median number of alarm noises per hour (monitor or ventilator) was not significantly different between day and night. Conversely, the median number of conversation noises per hour was statistically higher during the day than during the night (28 [10–53], vs. 5 [4–12, 15, 16, 21], *p* = 0.02).

### Impact of sound intensity on sleep

**Fig. 2:**
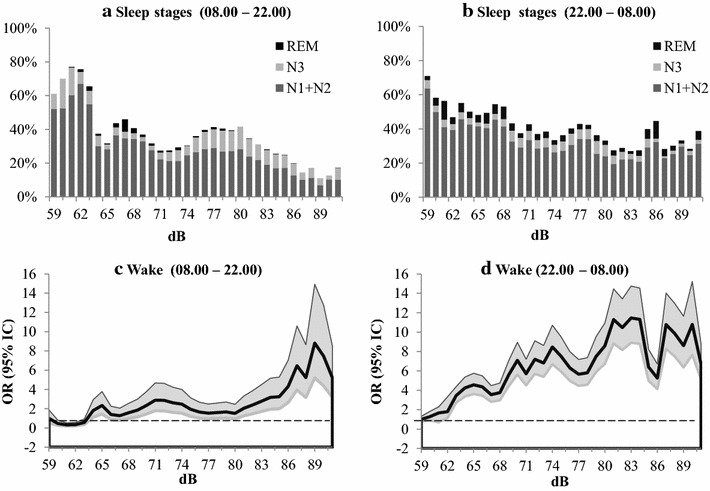
Sleep and wakefulness as a function of sound level. *Above* respective percentages of sleep stages for each median sound level for day time (08.00–22.00) and night time (22.00–08.00) are shown. *Below* OR (95% IC) risk of triggering wakefulness from sleep as a function of noise median levels is shown for day time (08.00–22.00) and night time (22.00–08.00)

A significant increase (95% CI > 1) in waking time was observed with a cutoff sound greater than 63 dBC during the day and greater than 59 dBC at night (Fig. 2). Figure 2a, b shows the median percentage of each sleep stage for each median sound level for day and night periods. N3 and REM sleep occurred even when the sound intensity levels were greater than 80 dBC at night. During daytime, there was almost no REM sleep at sound levels greater than 80 dBC; however, N3 sleep persisted. Figure 2c, d shows the OR of triggers for awakening from sleep during the day and during the night. The curve profiles are very different for the day and the night, with a later and clear cutoff profile above 63 dBC during the day, and a lower and progressive OR increase above 59 dBC during the night.

The multinomial analysis of the effect of sound intensity levels on sleep stages is reported in Table 4. There was a globally significant effect of sound on N1, N2 and REM sleep whatever the period (night or day) but not on N3.

**Table 4:** Multinomial logistic regression between sleep stage and sound (dBC)

	Night (22h00–08h00)	Day (08h00–22h00)
	OR (95% CI)	OR (95% CI)
Awakenings	1.0 (–)	1.0 (–)
N1	0.93 (0.93–0.94)***	0.97 (0.96–0.97)***
N2	0.95 (0.94–0.96)***	0.97 (0.96–0.97)***
N3	1.01 (1.00–1.02)	1.00 (0.99–1.01)
REM	0.91 (0.88–0.91)***	0.96 (0.95–0.97)***

*OR* (*95% CI*) odds ratio and 95% confidence interval

* *p* < 0.05; *** *p* < 0.001

**Fig. 3:**
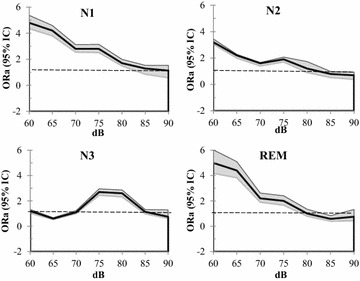
Multinomial adjusted OR (ORa 95% CI) for each sleep stages as a function of sound levels. OR are adjusted for hours and subjects

Adjusted ORs (ORa) for each sleep stages as a function of sound levels are shown in Fig. 3. Sound intensity was significantly linked to waking (95% CI > from N1–N2 and REM sleep when the level was below 80 dBC and from N3 when the level was below 85 dBC). Logistic regression analyses showed that sound levels impacted significantly the occurrence of waking (*p* < 0.001), arousals (*p* < 0.001) and sleep-to-wake transitions for N2 to waking (see Table 5). No specific predictive awakening effect of sound level was observed for N1, N3 or REM sleep stages. The OR for arousals becomes significant (95% IC > 1) when sound intensity level was above 77 dBC.

**Table 5:** Logistic regression between dependent variable and sound levels (dBC)

Dependant variable	Coefficient	SD	Wald statistics test	OR (95% CI)	Likelihood ratio test
Waking	0.049	0.009	1965***	1.05 (1.04–1.05)	206***
Arousal	0.018	0.004	15.4***	1.02 (1.01–1.03)	15.0***
Transitions	−0.028	0.004	58.8***	0.97 (0.96–0.98)	61.6***
N3 to W	0.052	0.031	1.7	1.05 (0.99–1.12)	2.7
N2 to W	0.048	0.009	27.6***	1.05 (1.03–1.07)	26.2***
N1 to W	−0.014	0.02	0.6	0.99 (0.95–1.02)	0.6
REM to W	0.066	0.05	1.8	1.07 (0.98–1.17)	1.6

*OR* (*95% CI*) odds ratio and 95% confidence interval

*** *p* < 0.001

Qualitatively, all of the sound sources had a significant impact on sleep stage transition and on arousal. However, ventilator alarms had the highest impact: 7.4 (6.1–8.7) on arousals in daytime and 10.9 (9.8–11.9) at night (Table 6).

**Table 6:** Influence of sound sources on sleep stages transitions and arousals. (Supplementary material)

	Day (08h00–22h00) ORa (95% IC)	Night (22h00–08h00) ORa (95% IC)
Alarm, *n*/h	1.9 (1.5–2.4)	1.9 (1.5–2.4)
Mechanical respirators, *n*/h	2.6 (2.3–2.9)	1.8 (2.3–2.9)
Talking; staff conversations, *n*/h	2.0 (1.6–2.4)	2.0 (1.6–2.4)
Other, *n*/h	1.9 (1.5–2.4)	1.8 (1.8–3.2)
Arousals		
Alarm, *n*/h	4.9 (3.4–6.7)	9.8 (8.6–11.3)
Mechanical respirators, *n*/h	7.4 (6.1–8.7)	10.9 (9.8–11.9)
Talking, staff conversations, *n*/h	3.0 (2.4–3.6)	5.2 (4.5–6.1)
Other, *n*/h	3.8 (2.3–6.3)	8.4 (6.9–10.1)

*ORa* OR adjusted for subject, hour and age

## Discussion

Knowledge about the impact of sound intensity in ICU is very limited, and our study aims to analyze sleep and sound objectively. A high prevalence of noise complaints from ICU patients has been reported in several previous studies [12–14, 22, 23]. Poor sleep is considered a major concern in the ICU because of its potential interaction with other psychological and somatic diseases and its impact on rehabilitation [14, 22, 23].

Sleep may be disturbed by multiple factors in ICU patients: pain [24, 25], high temperature [26], lighting [27], stress [24, 25], metabolic functions [28], the impact of mechanical ventilation [24, 29], adverse effects of some medications [30], and also by noise [31, 32]. However, it is unclear which sources of sound intensity are linked to the pathogenesis of sleep disturbance in ICU [33].

Using our miniaturized multi-channel ambulatory recording device, we succeeded in recording appropriately the different parameters necessary to score sleep in 30-s intervals in all our subjects. This miniaturized system is better tolerated than other standardized and larger PSG systems previously used in ICU and the shorter electrodes reduce interference.

Overall this study showed a fairly normal TST in subjects aged 64.2 ± 13.6 years—with a median of 502.2 [283.2–718.9] min and with a median nocturnal sleep of 356.9 min [188.6–590.9] and median daytime sleep of 168.5 [142.5–243.3] This TST is quite similar to the natural level (around 360 min per night) found in a meta-analysis of 65 PSG studies including 3577 healthy subjects (ranging from 5 to 102 subjects, mean age 65 years old) [33]. Of note, this meta-analysis gave no reference to 24-h sleep, which has only been estimated subjectively by epidemiological surveys at around 440 min in the general adult population [33].

Twenty-four hour TST is arguably a better measurement to consider in ICU patients as they are on continuous bed rest (except when activated or mobilized by staff) and their sleep may not follow a mono-episodic profile. In our group as mentioned above, patients slept an average of 6 h at night, but also a median of 2.5 h during the day (168.5 min [142.5–243.3]). One previous study assessed day and night sleep over 24 h and found an altered circadian rhythm [23]. Assessing 24 h sleep is crucial to a better understanding of the impact of sleep on ICU prognosis as subjects clearly take daytime naps and promoting sleep in ICU patients may take advantage of this opportunity.

Sleep quality was disturbed more than sleep quantity in our patients. The median N3 percentage was 6.5% [0–23.6] compared to 26.1% [34], and median REM sleep 3.9% [0–10.1] vs. 20% [34]. This poor quality has been reported previously [10, 35, 36]. In our study, we observe a significant link between sleep changes and arousals and noise, suggesting a partial explanation for lack of N3 and REM sleep.

REM sleep represented only 4% of TST during the 24 h, which is lower than the usual rate in normal sleepers (15–20%) [3]. N3 sleep represented 9% of TST, also lower than the usual percentage in normal sleepers (15–20%) [2].

We used sleep analysis according to the American Academy Sleep of Medicine (AASM). Other studies have shown that AASM was often an insufficient monitor for sleep stages in critically ill patients. Therefore, a modified AASM has been proposed by Watson et al. which can be used in critically ill patients [37]. However, in our study, all the sleep stages in these ventilated patients could be pre-categorized as REM or non-REM, N1, N2, N3 and awake. No atypical patterns were observed, and absent signal was only 0.2%. One possible explanation for this finding could lie in the category of patients: the 11 patients included in the present study were highly selected. They were non-sedated, undergoing weaning and post-resolution of disease. In other words, they were long-term ventilated patients without a cute illness. They were ventilated and in the ICU, but not critically ill. The fact that these patients were not critically ill might be an explanation for the lack of atypical sleep patterns. Secondly, the ActiWave itself, with electrodes positioned close to the sleeping brain, may have delivered more accurate quality of sleep signals than previous studies.

Heightened sound intensity levels are considered to be a major sleep-disturbing factor, whatever the context. Sound levels in hospital should not exceed 35 dBA LAeq in areas where patients are being treated or observed, with a corresponding LAmax of 40 dBA [38].

Previous studies have already observed that sound is a significant sleep disturber in ICU [29, 33], but the authors [29, 33] reported that this was only responsible for <20, and 17% of awakenings. Our study shows that sound levels above 77 dBC are associated with awakenings 60% of the time during the night (Fig. 2b). The median noise level at night (70.2 dB [65.1–80.3]) is considerably greater than the WHO limit, and sound levels >59 dBC at night (10 pm–8 am) and >63 dBC during the day were significantly linked with awakenings in ICU patients. Interestingly, monitor alarms and mechanical ventilator alarms, integral to the ICU environment and used for patient safety, also significantly disturbed sleep. Currently, there are suggestions that “noise-alarms” be replaced with other emergency signals such as light at nursing stations. Our study only demonstrates the negative aspect of the “noise-alarm”. This study identifies that these ICU and sound levels are above the limit recommended by the WHO and result in a higher incidence of disturbance of sleep patterns. Apart from alarms, other sounds coming from people around are implicated in the discussion of sleep continuity and quality.

We acknowledge that there are limitations to our study. For example, the evidence is based on a single 24-h PSG recording. It is known that the first PSG night is more disturbed than regular sleep, and two nights are required for pharmaceutical trials. Also we did not take into account the light environment of ICU rooms. Light at night is also known to alter the biological clock cycle and to disturb sleep. We recognize that concentrating on light and sleep in ICU is also a major issue in ICU [27, 29]. In our study, we did not assess the circadian factors that may influence the biological clock. Further research could record wrist actigraphy data from patients over a longer period.

Another potential limitation is that our study was limited to a group of 11 subjects who did not represent all patients hospitalized in the ICU. They had not received sedative medication recently and were not critically ill. However, in this preliminary study we deliberately concentrated on patients with an average of 22.1 ± 18.5 days stay in ICU, avoiding patients in the immediate post-diagnosis emergency period. We believe that our results reinforce and extend previous understanding of the fact that poor sleep is a persistent problem in ICU patients and that this is partly due to the negative effects of the immediate environment. Improving the physical and sensory aspects of the ICU environment represents an important and still under-researched potential for improving the sleep quality, and therefore the rehabilitation potential, of vulnerable patients.

## Conclusions

In summary, our work shows, in a small sample of 11 non-severe ICU patients, that AW combined with T3 are good tools to record sleep and noise throughout a 24-h period in ICU patients. Sleep continuity is disturbed by alarms, particularly those of mechanical ventilators and monitors in ICU patients who are ventilated. Our study supports the need to evaluate alarms and emergency signaling in ICU. We found that 60% of awakenings are triggered by a noise higher than 77 dBC. However, further research is needed in order to be able to apply our conclusions to a wider population of ventilated critically ill patients.

## Abbreviations

AASM: American Academy of Sleep Medicine
APACHE: acute physiology and chronic health evaluation scoring
BMI: body mass index
EEG: electroencephalogram
EOG: electrooculogram
EMG: electromyogram
ICU: intensive care unit
N1: non-REM sleep stage 1
N2: non-REM sleep stage 2
N3: non-REM sleep stage 3
OSAS: obstructive sleep apnea syndrome
PEEP: positive end-expiratory pressure
R: REM sleep
SAPSII: simplified acute physiology score
TST: total sleep time
